# Reliability and discriminatory power of methods for dental plaque quantification

**DOI:** 10.1590/S1678-77572010000200014

**Published:** 2010

**Authors:** Daniela Prócida RAGGIO, Mariana Minatel BRAGA, Jonas Almeida RODRIGUES, Patrícia Moreira FREITAS, José Carlos Pettorossi IMPARATO, Fausto Medeiros MENDES

**Affiliations:** 1 DDS, MSc, PhD, Department of Pediatric Dentistry, School of Dentistry, University of São Paulo, São Paulo, SP, Brazil.; 2 DDS, PhD, Department of Pediatric Dentistry, School of Dentistry, University of São Paulo, São Paulo, SP, Brazil.; 3 DDS, MSc, PhD, University Cruzeiro do Sul (UNICSUL), São Paulo, SP, Brazil.; 4 DDS, MSc, PhD, Department of Restorative Dentistry, School of Dentistry, University of São Paulo, São Paulo, SP, Brazil.

**Keywords:** Dental plaque, Biofilms, Fluorescence, Visual indices, Camera

## Abstract

**Objective:**

This *in situ* study evaluated the discriminatory power and reliability of methods of dental plaque quantification and the relationship between visual indices (VI) and fluorescence camera (FC) to detect plaque.

**Material and Methods:**

Six volunteers used palatal appliances with six bovine enamel blocks presenting different stages of plaque accumulation. The presence of plaque with and without disclosing was assessed using VI. Images were obtained with FC and digital camera in both conditions. The area covered by plaque was assessed. Examinations were done by two independent examiners. Data were analyzed by Kruskal-Wallis and Kappa tests to compare different conditions of samples and to assess the inter-examiner reproducibility.

**Results:**

Some methods presented adequate reproducibility. The Turesky index and the assessment of area covered by disclosed plaque in the FC images presented the highest discriminatory powers.

**Conclusions:**

The Turesky index and images with FC with disclosing present good reliability and discriminatory power in quantifying dental plaque.

## INTRODUCTION

Methods for dental plaque assessment have been extensively employed in researches of periodontal diseases^4,17,20^, dental caries^8,17,21,24^, and in evaluating efficacy of oral hygiene products^5,9,15,16,18^. Most studies on dental plaque indices have focused in periodontal issue^4,19,20,23^, but some studies have also shown the association of plaque with dental caries^8,15,22,25^.

In order to improve the quality of research in this field, methods of plaque quantification should have good discriminatory power and reliability. Some appropriated indices to assess the association of plaque with periodontal disease have presented good reproducibility^5,12^, and few manuscripts have demonstrated their discriminatory validity^1,19^. However, there is still no research on the evaluation of the feasibility of methods for quantifying the dental plaque formed under high frequency of sucrose exposition. This kind of plaque is probably more prone to provoke dental caries^13^. Therefore, studies should be conducted to test the reliability and discriminatory power of methods of plaque quantification in these conditions.

Moreover, recent studies have demonstrated that plaque fluoresces in red when it is excited by light with a wavelength peak of approximately 405 nm emitted from the Quantitative lightinduced fluorescence (QLF) device^7,16,17,25^. This red autofluorescent plaque has been related to mature plaque^7,17,25^. Recently, a novel fluorescence camera (FC) using a similar wavelength (Vista Proof, Dürr Dental, Bietigheim- Bissingen, Germany) was recently introduced into the market^22^. However, it uses another kind of software to exhibit the captured images, what could imply in different analysis mode in the identification of mature plaque. Another method to distinguish mature from immature plaque is the two-tone disclosing agent, which stains the mature plaque in blue purple and the immature plaque in red3,11. Indeed, a visual index has previously been described in order to make this kind of distinction5. Nevertheless, comparison between this visual index and the laser fluorescence camera in detecting mature plaque has not been assessed yet.

The aim of this *in situ* study was to evaluate the reliability and discriminatory power of visual methods using two-tone dye and laser fluorescence camera in quantifying dental plaque formed under high frequency of sucrose exposition. It was also verified whether the presence of dental plaque showing red autofluorescence with the FC could be correlated with the plaque stained in blue purple with the two-tone disclosing dye. The null hypothesis tested was that there is no difference among methods regarding evaluation of dental plaque and reliability.

## MATERIALS AND METHODS

This *in situ* study was approved by the local Research Ethics Committee, and volunteers’ written consent was obtained.

### Sample and appliances preparation

Forty bovine incisors were selected, cleaned with rotating bristle brush and pumice/water slurry, and washed with tap water. Then, enamel blocks of 4 x 4 x 2 mm were cut under refrigeration to avoid overheating and visually checked for the absence of cracks or defects. Thirty-six plane enamel blocks without defects were selected and sterilized with gamma radiation (25 kGy). The samples were stored in 100% humidity until the beginning of the study.

Subsequently, six healthy dentate volunteers (aged 25-34 years), PhD students, without currently active caries lesions or periodontal disease, were instructed to use removable acrylic palatal appliances containing six bovine enamel blocks placed in the palate. The appliances would be used by the volunteers all day, being removed only during the meals and toohbrushing. The volunteers performed oral hygiene with fluoridated dentifrice (1,500 ppm), three times a day, during experimental phase. The appliances were out of oral cavity up for one hour/day.

**Figure 1:**
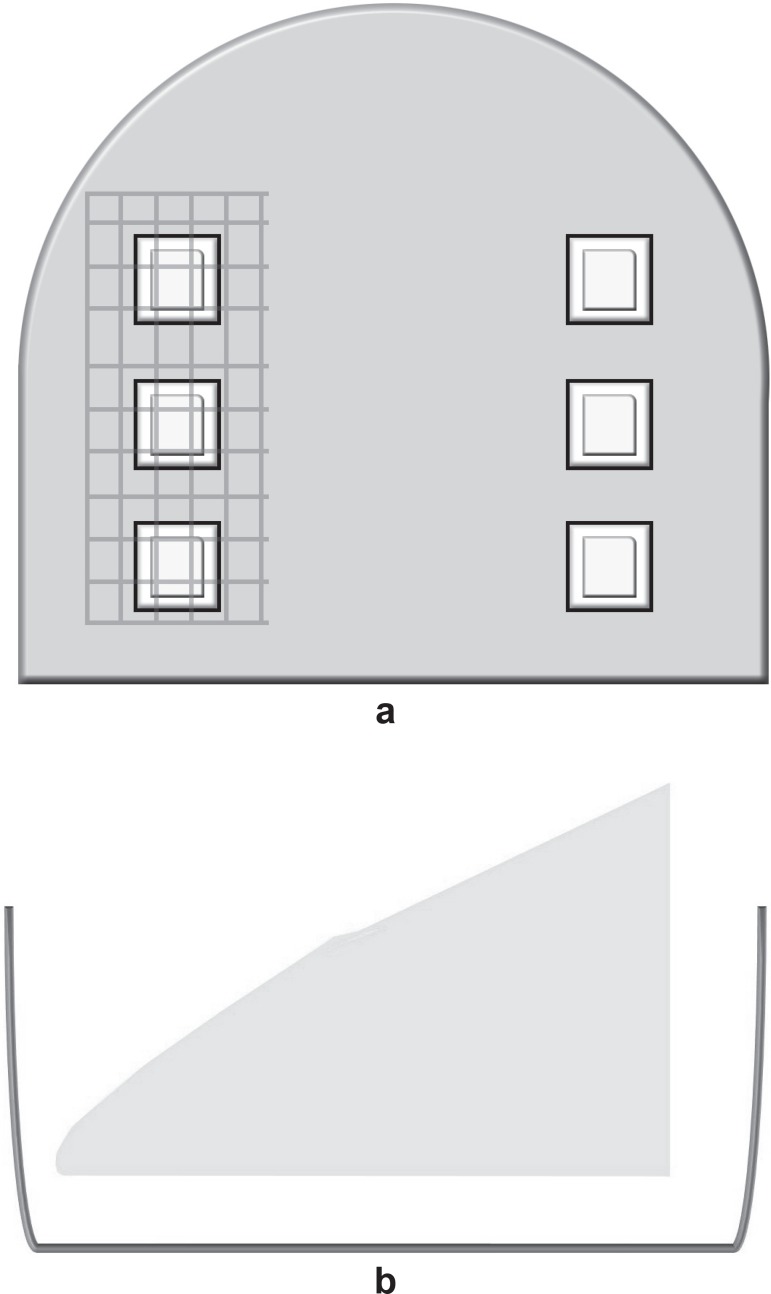
Palatal appliance used in the *in situ* study. (a) Schematic drawing - six blocks: three protected with a plastic mesh and three with no protection; (b) Position of the blocks inside the recess in the acrylic flange

The enamel blocks were located in a recess 1.0 mm below the acrylic flange and fixed using composite resin with an inclination of approximately 30 degrees, simulating a buccal surface. This position could guarantee the creation of regions with different predisposition to accumulation of dental plaque. On three blocks, a plastic mesh (0.27 mm thickness, 1mmx1mm squares, nylon monofil, Lauhman, Sumaré, SP, Brazil) was fixed with acrylic resin onto the acrylic surface to protect the enamel blocks. The other three blocks were not protected with the plastic mesh. This procedure was done in order to permit higher plaque accumulation on the covered enamel blocks than on the blocks without the plastic mesh (Figure 1).

The volunteers used the appliances during four days, and they were oriented to drip 20% sucrose solution eight times *per day*^24^. After four days, each volunteer had their plastic mesh removed, and two enamel blocks (one previously protected by the mesh and another without protection) were randomly selected and cleaned with pumice/ water slurry and rinsed with water by one of the researchers (MMB). This procedure was performed to simulate one condition of high plaque accumulation (with the plastic mesh), another with low plaque accumulation (without the plastic mesh) and the third group without plaque accumulation (that undergone to professional cleaning).

### Dental plaque assessment

Two other examiners (DPR and FMM) assessed the blocks in order to quantify the plaque accumulation using different methods. The examiners were not aware about the position, protection, and sample numbering, or cleaning procedure of each block. The order of samples evaluation was randomized for each method by the one who performed the cleaning of the samples. The examinations were performed independently, and the examiners were unaware of each other’s results or the results obtained with other methods. In order to simulate the cervical margin, the examiners were oriented to consider the specific inclination of the block as cervical margin.

In the assessment of plaque accumulated on the blocks, the following visual indices were used: Silness and Löe index (visible plaque), Turesky index (disclosed plaque) and Ekstrand index (disclosed plaque in two-tone dye). The indices were introduced to the examiners, but no training or calibration session was conducted. Images using the FC were taken without and after disclosing. The Turesky index was employed in the evaluation of these images.

Firstly, the examiners used the Silness and Löe visible plaque index^20^ to assess the amount of plaque on each enamel block: 0: no visible plaque; 1: plaque detectable only with a probe; 2: a thin layer of plaque gingival area; 3: great accumulation of plaque.

However, as probing was not done to avoid disturbing the plaque, in order to maintain to further evaluations with the disclosing agent, the score 1 was not coded in this assessment.

After, the examiners took images of the blocks using the FC (Vista Proof, Dürr Dental, Bietigheim-Bissingen, Germany) device. The images were firstly taken without any plaque disclosing dye, with standardized distance (0.5 cm from tooth surface, with a spacer) and they were recorded in the software recommended by the manufacturer. Then, a two-tone disclosing dye (Replak, Dentsply, Rio de Janeiro, Brazil) was used according to the manufacturer’s instructions and the examiners coded the plaque amount on the enamel blocks using the index devised by Quigley and Hein^18^ modified by Turesky:^23^: 0: no plaque; 1: separate flecks of plaque at the cervical margin; 2: a thin continuous zone of plaque (up to 1 mm) at cervical margin; 3: a zone of plaque wider than 1 mm but covering less than 1/3 of crown; 4: plaque covering at least 1/3 but less than 2/3 of the crown; 5: plaque covering more than 2/3 of the crown.

Then, the examiners assessed the blocks using a modification of Ekstrand index to evaluate the plaque status^8^: 0: no plaque; 1: plaque stained in red (immature plaque); 2: plaque stained in blue purple (mature plaque).

New images were taken with the FC, but with the plaque disclosed. In addition photographs were taken using a digital camera (CEOS Digital Rebel XTi; Canon Inc., Tokyo, Japan), maintaining standard distance from the block and light source.

The examiners used the Turesky index in the images obtained with the FC, with and without the disclosing agent. After that, the images were analyzed independently by two examiners using the image analysis software (Leica Qwin, Leica Microsystems, Heidelberg, Germany) to evaluate the area of the blocks covered by plaque. Firstly, examiners detected the area of the entire block, and after, the area covered by plaque. The result was obtained as percentage covered by plaque. This kind of analysis was done with the images obtained with the FC with and without the disclosing agent, as well as with the photographic images obtained with the digital camera. The automatic detection of the area covered by plaque using the software was not employed.

In order to evaluate the association between red autofluorescence of plaque using the FC device and the dental plaque disclosed in blue purple with the two-tone dye, one examiner (FMM) evaluated the area covered by red fluorescent plaque assessed using the FC device without disclosing, and the area disclosed in blue purple in digital photographs.

### Statistical analysis

The percentage of area covered by plaque obtained with the different methods did not show a normal distribution by the D’Agostino Pearson test. To evaluate the discriminatory power, the methods should be able to distinguish the following predictions: 1-enamel blocks which were cleaned present less dental plaque than the blocks without any cleaning procedure; 2-samples without the plastic mesh protection have a higher amount of plaque than cleaned blocks, but less than the blocks protected by the plastic mesh. Thus, the methods of plaque quantification should reflect these differences according to the different specimens’ conditions. To compare the differences among the groups, a Kruskal-Wallis test was employed for all methods, and post hoc comparisons were performed using Bonferroni test.

The inter-examiner reproducibility with the indices was firstly evaluated using a Cohen’s Kappa test,^6^ and quadratic weighted Kappa test.^10^ For the methods that evaluated the area covered by plaque, the inter-examiner reliability was calculated using the intraclass correlation coefficient (ICC) and 95% Confidence Interval (95% CI). In order to compare the values obtained with the indices, the results were divided in a 5-point scale according to the quintiles, and Cohen’s Kappa^6^ and weighted Kappa values^10^ were calculated.

The correlation between the area of red autofluorescence of dental plaque using the FC and area disclosed in blue purple with the twotone agent was expressed with Spearman correlation coefficient (Rs) and 95% CI. To compare the means obtained by both methods, a Wilcoxon test was used. The level of significance for all the tests was chosen as p < 0.05 and the software was MedCalc 9.3.7.0 (Medcalc, Mariarke, Belgium).

## RESULTS

The discriminatory power of the different methods for dental plaque quantification is presented in Table 1. All methods showed difference at least among two groups. However, the Turesky index and the percentage of area covered by plaque evaluated using the FC after disclosing demonstrated differences among the three groups, showing better discriminatory power than the other methods (Table 1).

**Table 1:** Discrimination among different conditions of the enamel bovine samples evaluated with different methods of plaque quantification

	**Cleaned**	**Not Cleaned**	
		** Without plastic mesh **	**With plastic mesh**
Silness & Loe *	0.17 ± 0.56ª	0.29 ± 0.81ª	2.04 ± 1.04^b^
Turesky *	0.96 ± 0.20ª	1.88 ± 1.39^b^	3.58 ± 1.61^c^
Ekstrand *	1.33 ± 0.56ª	1.17 ± 0.38ª	1.79 ± 0.41^b^
FC + Turesky *	1.58 ± 0.88ª	1.18 ± 0.70ª	3.13 ± 1.33^b^
FC + disclosing + Turesky *	1.63 ± 0.88ª	2.21 ± 1.41ª	3.92 ± 1.18^b^
FC **	0.66 ± 2.09ª	0.16 ± 0.11ª	0.41 ± 0.17^b^
FC + disclosing **	0.34 ± 0.11ª	0.43 ± 0.18^b^	0.57 ± 0.19^c^
Photographic images **	0.35 ± 0.11ª	0.52 ± 0.24^b^	0.61 ± 0.29^b^

Different letters indicate statistically significant differences among the groups within the same row (p < 0.05).

* Mean of scores standard deviations of each index.

** Mean of percentage area of block surface covered by plaque ± standard deviations. FC = Fluorescence camera.

Regarding the reliability, the three indices presented similar inter-examiner reproducibility using Cohen’s Kappa analysis, but the value of Turesky index was improved when the weighted approach was used (Table 2). When the Turesky index was used in the FC images with or without disclosing, the values were lower than those obtained in the assessments for the samples directly. Concerning the methods of quantification of the area covered by dental plaque, the method using the FC after disclosing presented higher ICC value than the method using FC without disclosing and the method with the digital photographic images. After dividing the percent area values into quintiles, the crude Kappa results of three methods were lower than those obtained with the indices, but the FC method with disclosing showed inter-examiner reproducibility value similar to Turesky index using the weighted Kappa test (Table 2).

**Table 2:** Inter-examiner reliability values obtained with different methods of plaque quantification

	**Cohen's Kappa (SE)**	**Weighted Kappa (SE)**	**ICC (95 % CI)**
Silness & Loe	0.625 (0.127)	0.664 (0.160)	
Turesky	0.621 (0.118)	0.780 (0.106)	
Ekstrand	0.624 (0.128)	0.646 (0.163)	
FC + Turesky	0.248 (0.112)	0.513 (0.159)	
FC + disclosing + Turesky	0.127 (0.097)	0.633 (0.155)	
FC	0.247 (0.100) *	0.654 (0.156) *	0.700
			(0.526 - 0.819)
FC + disclosing	0.481 (0.102) *	0.785 (0.165) *	0.881
			(0.794 - 0.933)
Photographic images	0.348 (0.103) *	0.592 (0.160) *	0.764
			(0.600 - 0.867)

* Calculated after division into a 5-score scale according to the quintiles. ICC= intraclass correlation coefficient; FC = Fluorescence camera; SE = Standard error; CI = Confidence interval.

The area of red autofluorescence of dental plaque observed using the FC was correlated to the area of dental plaque disclosed in blue purple with the two-tone disclosing dye (Rs = 0.727; 95% CI = 0.524 - 0.852, p < 0.0001). However, the area of plaque disclosed in blue purple (mean = 0.297) was statistically significant higher than the area of red autofluorescent plaque (mean = 0.216, p = 0.0008).

## DISCUSSION

The majority of studies that assessed the feasibility of methods for dental plaque quantification emphasize its relationship to periodontal disease^1,4,5,9,12,19,20^. No previous research has evaluated the dental plaque induced in a challenge with a high frequency of sucrose exposition. Indeed, an *in situ* model was used to simulate this condition, once this cariogenic challenge could not be reproduced clinically for ethical reasons. As the visual methods employed were not proposed to be used in square blocks, but in dental surfaces, the specimens were positioned in the removal appliance aiming to mimetic different regions of a dental surface. Thus, we assessed the power discrimination and reliability of methods for dental plaque quantification under these conditions.

In this study, Turesky index and the quantification of the area covered by disclosed plaque detected by the FC device presented the highest discriminatory power. It was previously expected that the cleaned blocks had to present less amount of plaque than the other samples, and that the enamel blocks without plastic mesh protection had to show lower amount of plaque than the specimens protected by the plastic mesh. These two methods were able to demonstrate these differences. Other methods presented significant differences between two of three groups, but no difference within the three groups. Another study claimed that indices have presented better discriminatory power than measurement of area covered by plaque^1^, since the latter is unable to detect small differences in dental plaque quantity^17^, corroborating our findings. On the other hand, another study showed that the area assessment was better in detecting higher amount of plaque than other visual indices^19^.

The higher discriminatory power emphasizes the ability of these methods in distinguishing dental plaques in different amounts. The Turesky index scores the plaque amount according to the part of the dental surface in which the plaque is found. Therefore, smaller amounts of plaque (cleaned blocks) were usually associated with lower scores. Additionally, the disclosed plaque detected by the FC probably tended to be identified easily in specimens containing higher amount of plaque than in those previously cleaned. A possible explanation for lower discriminatory power of some indices could be because of the low number of scores (Ekstrand index, for instance). Exclusion of score 1 of Silness and Löe index could explain its moderate performance.

Regarding the reliability, previous studies have demonstrated good intra- and inter-examiner agreement when both indices and planimetric methods were used.^12,14,17,21^ In the present study, raw Kappa values were low, but when the weighted Kappa test was employed, the interexaminer reliability presented higher values. In fact, this approach is more appropriated for ordinal scores^10^. In order to compare the methods of area measurement with the indices, the values were divided in a 5-point scale according to the quintiles, and the Cohen’s and Weighted Kappa tests were employed. After this division, the weighted values were similar to the values obtained with the indices. Furthermore, the two methods that presented the best discriminatory power also presented the highest inter-examiner reliability.

In earlier studies, previously to plaque assessment, the examiners were trained and calibrated^12,14,21^. If the training had been performed in our study, probably the agreement values would have been higher.

As the present study intended to evaluate plaque formed under high frequency of sucrose exposition, which is more related to caries lesions induction, Turesky index and measurement of area covered by disclosed plaque detected by the FC seem to be more indicated to assess dental plaque in studies of dental caries, since they presented good reliability and discriminatory power. It has to be stated that there is an increase in the cost, regarding the use of FC.

As visual index using two-tone dye and laser fluorescence devices were possibilities to identify mature plaque, the comparability between them is extremely important. A previous study evaluated the relationship between the assessment of mature plaque with a quantifying light fluorescence (QLF) and a typical one-tone dye^7^ However, no comparison between fluorescence devices and two-tone dyes were performed.

A red autofluorescence of the dental plaque illuminated by a blue light (408 nm) from the QLF device has been observed.^7,16,17,25^ Authors have suggested that the obligate anaerobic bacteria are the responsible for the red autofluorescence, and these bacteria are indicative of mature plaque^7,25^. In our study, we observed the red autofluorescence mainly in the plaque on the enamel blocks which were protected by the plastic mesh, in which it is probably that a more complex plaque has been formed. However, we did not evaluate microbiologically the plaque formed on the blocks, not even their mineral loss.

The method using the FC without disclosing dye did not reliably assess the dental plaque. However, when we employed the disclosing dye and took the images with the FC, the method was suitable. Other studies using an intraoral camera capable to obtain images and assess the plaque area have been performed, but with a regular illumination^15,21^. The advantage in using the novel FC device would be the possibility to achieve images of red autofluorescent plaque without any disclosing dye, which is a plaque possibly associated to high dental caries risk^25^, and further, to obtain images of the disclosed plaque. Despite of that, our study showed poor reproducibility in using evaluation of FC images without disclosing. The poor reproducibility per se could be considered an important disadvantage of the method. However, considering the lack of previous intensive training in using the device, this parameter can be improved if examiners are previously trained. Moreover, the FC could be used for dental caries detection after the cleaning of the teeth^22^, nevertheless, this was not the aim of our research. Therefore, more studies using the FC with dental plaque and caries evaluation are also necessary.

The FC seems to have the same principles of the QLF, but studies comparing both devices have not been performed yet. Considering the FC uses the same wavelength of the QLF, but is associated with different software, the simple extrapolation of results obtained with the first one was not really appropriated. The QLF device has already been extensively studied and previous researches have shown good results in detecting disclosed and undisclosed dental plaque.^16,17,25^ Furthermore, the QLF has shown good results for caries lesions assessment^2^.

Another method to differentiate mature from immature dental plaque is using a two-tone disclosing agent^3,11^.This dye stains the immature plaque in red and the old plaque in blue purple. In the present study, we observed a significant correlation between the area of dental plaque exhibiting red autofluorescence and the area stained in blue purple. However, the mean of area stained in blue purple was significantly higher than the mean area of red fluorescent plaque. This difference could be explained by the different mechanisms to detect mature plaque. While the two-tone disclosing dye detects mature plaque due to its thickness, the phenomenon of red fluorescence is probably due to some bacterial metabolites, possibly porphyrins. Additional studies relating mature plaque detected by both methods and increased risk of oral diseases must be carried out.

## CONCLUSIONS

Turesky index and the quantification of the area covered by plaque using the new FC after disclosing have good reliability and discriminatory power in quantifying dental plaque formed under high frequency of sucrose exposition. Furthermore, there are correlation between red autofluorescence and dental plaque disclosed in blue purple, but the method with two-tone disclosing agent shows a higher area of mature plaque than the fluorescence-based method.
